# Pathogenic Variant in* ACTB*, p.Arg183Trp, Causes Juvenile-Onset Dystonia, Hearing Loss, and Developmental Delay without Midline Malformation

**DOI:** 10.1155/2017/9184265

**Published:** 2017-04-12

**Authors:** Erin Conboy, Filippo Vairo, Darrel Waggoner, Carole Ober, Soma Das, Radhika Dhamija, Eric W. Klee, Pavel Pichurin

**Affiliations:** ^1^Center for Individualized Medicine, Mayo Clinic, Rochester, MN, USA; ^2^Department of Clinical Genomics, Mayo Clinic, Rochester, MN, USA; ^3^Department of Human Genetics, University of Chicago, Chicago, IL, USA; ^4^Department of Pediatrics, University of Chicago, Chicago, IL, USA; ^5^Department of Clinical Genomics and Neurology, Mayo Clinic, Phoenix, AZ, USA

## Abstract

*ACTB* encodes the *β*-actin, and pathogenic variations in this gene have typically been associated with Baraitser-Winter cerebrofrontofacial syndrome, a congenital malformation syndrome characterized by short stature, craniofacial anomalies, and cerebral anomalies. Here, we describe the third case with the p.Arg183Trp variant in* ACTB* causing juvenile-onset dystonia. Our patient has severe, intractable dystonia, developmental delay, and sensorineural hearing loss, besides hyperintensities in the caudate nuclei and putamen on the brain MRI, which is a distinct but overlapping phenotype with the previously reported case of identical twins with the same alteration in* ACTB*.

## 1. Background

Dystonia is a movement disorder that is caused by sustained or intermittent cocontraction of agonist-antagonist muscle groups leading to abnormal posturing and repetitive movement. These dystonic movements can be repetitive and can be worsened by any voluntary movement and in some cases an intercurrent illness. In general, the underlying genetic cause has been unequivocally identified in several inherited forms of dystonia that have the “DYST” designation and for many other more complex forms of dystonia [1].

Actin is one of the major cytoskeletal proteins that participates in important cellular functions such as muscle contraction, cell motility, and cell signaling [2]. Given its importance and interaction with many other proteins, it is highly conserved throughout evolution. There are six actin isoforms in vertebrates: *α*-skeletal muscle, *α*-cardiac muscle, *α*-aortic smooth muscle, and *β*-cytoplasmic, *γ*-cytoplasmic, and *γ*-smooth muscle [3]. *β*-actin is encoded by* ACTB*, and pathogenic variations in this gene have typically been associated with Baraitser-Winter cerebrofrontofacial syndrome (BRWS; OMIM 243310), a congenital malformation syndrome typified by short stature, craniofacial anomalies, and cerebral anomalies [4, 5].

We describe the third case with the p.Arg183Trp pathogenic variant in* ACTB* causing juvenile-onset dystonia. Our case has a distinct but overlapping phenotype with the previously reported case of identical twins with the same p.Arg183Trp alteration in* ACTB* [6]. Their features included cleft lip and palate, skeletal abnormalities, hearing loss, developmental delay, and juvenile-onset dystonia.

## 2. Case Presentation

Our patient first presented at our institution at the age of 13 years for evaluation of dystonia that had begun approximately two years earlier. His dystonia affected his right upper extremity and recently worsened in interference with activities of daily living. He also had a history of bilateral sensorineural hearing loss first noticed at 8 months of age. He had, per parental report, passed the newborn hearing screen. The patient was a male born at term to consanguineous Hutterite parents and was the product of an uncomplicated pregnancy. He had motor and language delays necessitating physical therapy. He needed speech therapy and his hearing loss required cochlear implants. He had a mild intellectual disability, but no regression of skills. His height and weight were normal for age, following along the 10th and 20th percentiles for age, respectively. On exam, he was nondysmorphic and had no abnormalities aside from the dystonia. He had the subsequent onset of bulbar dystonia after our initial evaluation and this progressed to velopharyngeal insufficiency and dysphagia. Given speech was affected by his worsening bulbar dystonia; it was difficult to assess if the patient had cognitive decline. Brain MRI was completed to investigate this new onset bulbar dystonia; although it was limited by cochlear implants, it revealed symmetrical FLAIR T2 hyperintensity in the bilateral caudate nuclei and subtle parenchymal T2 hyperintensity in the bilateral basal ganglia.

**Figure 1:**
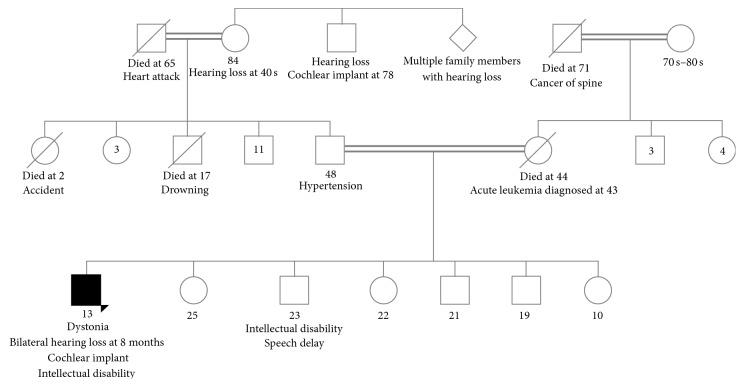
Family history showing the consanguineous union between the proband's parents and grandparents, 23-year-old brother with intellectual disability and speech delay, and grandmother and grand aunts and uncles with hearing loss.

Five siblings and his parents were healthy without evidence of hearing loss, dystonia, or intellectual disability (Figure 1). Genetic investigations included a chromosomal microarray, which demonstrated no clinically significant copy number changes, but several large regions of homozygosity, encompassing approximately 7% of the genome. A comprehensive dystonia next generation sequencing panel was sent to Medical Neurogenetics in 2014 and showed one variant of uncertain significance (VUS) in* PSEN1* and one VUS in* DDC*. The disorders associated with these genes do not have clinical overlap with our patient's phenotype and therefore were not thought to be relevant.

At the age of 15 years, the patient was admitted to the hospital for worsening and medically refractory dystonia. He underwent deep brain stimulator placement in his left motor cortex, which helped to alleviate his predominately right-sided dystonia. Unfortunately, this was of limited benefit and he eventually suffered from status dystonicus that necessitated a medically induced coma. He had a prolonged hospital course and died from complications of the worsening and uncontrolled dystonia.

## 3. Materials and Methods

### 3.1. Patient Consent

We evaluated the proband at the Mayo Clinic in Rochester, MN, USA. Father consented for collection of his and the proband's blood samples for analysis by Whole-Exome Sequencing (WES).

### 3.2. Genetic Analysis

WES of the proband was performed at Baylor Miraca Genetics Laboratory in Houston, Texas, USA. A detailed description of the method for exome sequencing and data analysis is published elsewhere [7]. Sanger sequencing was performed in the proband's and father's DNA for variant confirmation. Unfortunately, the mother of the proband was deceased, and we did not have access to mother's DNA at the time of exome sequencing. However, The University of Chicago Genetic Services Laboratory (Chicago, Illinois, USA) had a sample of the mother's DNA as a result of this family's participation in previous (unrelated) research and performed Sanger sequencing to confirm the variant in the proband and to test for its presence in both parents' DNA. DNA microsatellite analysis was also performed on the parental and proband samples to confirm that the correct parental samples were used. The parents and proband in this family had all previously provided consent for genetic studies. The* ACTB* cDNA reference sequence used was NM_001101.3.

## 4. Results and Discussion

WES of the proband detected a heterozygous c.547C>T (p.Arg183Trp) variant in the* ACTB* gene that was not present in the father. We confirmed that the mutation was a de novo alteration by sequencing mother's DNA, stored at the University of Chicago laboratory. This variant has not been observed in approximately 6,500 individuals of European and African American ancestry in the NHLBI Exome Sequencing Project (ESP) or in over 60,000 individuals in the Exome Aggregation Consortium (ExAC) or in the whole genome sequences of 98 Hutterites [8]. The variant was classified as deleterious by different in silico prediction tools. The same variant has been previously published in monozygotic twins that were diagnosed with deafness at the age of 4 years and DOPA-unresponsive dystonia and cognitive decline after 12 years of age [9]. The twins died in their early twenties secondary to dystonia associated aspiration pneumonia. They initially presented with cleft lip and palate, achalasia at the age of 2 years, and mild developmental delay. One twin developed cataracts at age 3, but the other had unaffected vision.* ACTB* was implicated as associated with the phenotype after postmortem brain examinations revealing abundant proteinaceous inclusions that stained positively for actin and cofilin, the actin regulatory protein [6]. The heterozygous gain-of-function pathogenic variant p.Arg183Trp was functionally validated by biochemical and morphological studies in addition to molecular modeling [9] confirming that it caused the twins' phenotype. Furthermore, a different group performed additional biochemical studies and molecular dynamics simulations that showed the change at residue 183 perturbs nucleotide release from actin monomers and polymerization behavior by inducing a closed state conformation of the protein [10].

Dystonia is a rare clinically and genetically highly heterogeneous movement disorder characterized by sustained or intermittent muscle contractions. Primary dystonia has an estimated prevalence of 16 in 100,000 people worldwide [11]. Dystonic symptoms can present from early infancy to late adulthood and can be classified as focal, multifocal, or generalized depending on the body parts affected. The adult-onset focal is the most prevalent form, with a positive family history reported in approximately 25% of the patients [12]. Inherited dystonias are the ones where a genetic etiology can be identified. Up to now, more than 200 genes have been linked to the different forms of dystonia [13], so massive parallel sequencing approaches, such as multigene panels or WES, are useful tools in the differential diagnosis of the affected patients. Our patient firstly presented with a focal evolving to multifocal and then a generalized form of a DOPA-unresponsive dystonia in a short period of time, making the gene by gene testing a more costly and time-consuming approach.

*ACTB* is also associated with BRWS, a malformation syndrome characterized by short stature, hypertelorism, bilateral ptosis, ocular colobomata, metopic ridging, and cerebral anomalies [4]. Recently, a group published the delineation of the mutation and phenotypic spectrum in 42 cases with BRWS [5]. Although there is marked phenotypic variability within this cohort, none of the patients manifested neurodegeneration or dystonia, features so far only associated with the p.Arg183Trp pathogenic variant. Also, our patient does not have any dysmorphic features associated with BRWS.

## 5. Conclusion

The previously reported twins with same p.Arg183Trp mutation as our patient had midline facial defects and skeletal anomalies; however our patient did not display these features of the phenotype. Common aspects of the phenotype shared between all three patients include developmental delay, mild intellectual disability, medically refractory dystonia with an especially severe bulbar component, and hearing loss.

WES is useful for identification of rare neurodegenerative disorders without well-established phenotypes. We report on a patient that harbors an already published de novo variant in* ACTB* with severe, intractable dystonia, developmental delay, sensorineural hearing loss, and brain changes on MRI significant for hyperintensities in the caudate nuclei and putamen.
